# Maternal and Perinatal Outcome of Maternal Obesity at RSCM in 2014–2019

**DOI:** 10.1155/2021/6039565

**Published:** 2021-02-09

**Authors:** Junita Indarti, Sulaeman Andrianto Susilo, Purnomo Hyawicaksono, Jimmy Sakti Nanda Berguna, Galuh Anindya Tyagitha, Muhammad Ikhsan

**Affiliations:** Department of Obstetrics and Gynecology, Dr. Cipto Mangunkusumo National General Hospital Jakarta, Pangeran Diponegoro Street 71, Jakarta, Indonesia

## Abstract

Obesity is a pandemic found in many countries. It is estimated that, in 2025, more than 21% of women in the world will suffer from obesity and its number keeps increasing yearly. Obesity in pregnancy is one of the important challenges in obstetric services given the prevalence and potential adverse effects on the mother and fetus. Obese women have a higher risk of developing gestational diabetes, gestational hypertension, preeclampsia, venous thromboembolism, postpartum hemorrhage, cesarean delivery, and maternal death. The aim of this research is to determine the prevalence of maternal and perinatal complication in various obesity grades. This research was an observational descriptive study using the cross-sectional design. The inclusion criterion is obese pregnant women whose delivery was done in Dr. Cipto Mangunkusumo National General Hospital (RSCM) from 2014 to 2019. The exclusion criterion in this study is the incomplete medical record. A total of 111 subjects were included in the study. Obesity grades in this study were based on World Health Organization (WHO) obesity, divided into 3 classifications which are obese I (30–34.9 kg/m^2^), obese II (35–39.9 kg/m^2^), and obese III (≥40 kg/m^2^). Maternal outcomes in this study were birth method, gestational diabetes, preeclampsia, and premature rupture of membrane (PROM). Perinatal outcomes in this study were preterm birth, birth weight, APGAR score, and postdelivery neonatal care. In this study, obese patients had a mean age of 31.23 years, mean gravida 2, parity 1, and abortion 0. Most of these patients used an intrauterine device (IUD) for family planning (74.8%). There were no differences in age, parity status, and family planning methods in each group of patients with different body mass index (*p* > 0.05). Maternal characteristics are the majority of deliveries performed cesarean delivery (86.5%), cases of diabetes mellitus are more common in obese I patients (50%), preeclampsia is more prevalent in obese grade II patients (34,4%), and premature rupture of membranes (PROM) is more common in patients with obese II (52,4%). However, there was no difference in the prevalence of maternal outcomes between groups. There was a median gestational age of 37 weeks in all obesity grades, the highest percentage of preterm births owned by obese II patients (32,6%), the mean birth weight of babies tends to increase along with the weighting of the body mass index group, and neonatal intensive care unit (NICU) treatment rooms were mostly occupied from mother with obese II groups (18%). There was no difference in the first-minute and fifth-minute APGAR scores between study groups (*p* > 0.05). There were no differences in perinatal outcomes between groups. There were no significant differences in maternal and perinatal outcomes prevalence between different obesity grades. However, the rate of maternal and perinatal complications in obese women is higher than the normal population, thus requiring sophisticated prevention and approach toward handling the pregnancy.

## 1. Introduction

Obesity in pregnancy is one of the important challenges in obstetric services given the prevalence and potential adverse effects on the mother and fetus. Obesity in pregnancy is a high-risk obstetric condition that requires special attention [1]. Obesity is a pandemic problem found in many countries. It is estimated that, in 2025, more than 21% of women in the world will suffer from obesity. In United Kingdom (UK), the prevalence of obesity in pregnancy rose from 9–10% in the early 1990s to 16–19% in the 2000s [2]. In the Indian subcontinent, the prevalence of obese or overweight married women (15–49 years) rose from 11 to 15% in 2005–2006 as per the National family Health Survey (NFHS) 3 and further to 20.6% as per NFHS 4. In 2014, an estimated 326,900 individuals were pregnant with obesity in Indonesia. In RSCM, the prevalence of pregnancy with obesity is 1% [1–4].

Obese women have a higher risk of developing gestational diabetes mellitus, gestational hypertension, preeclampsia, venous thromboembolism, postpartum hemorrhage, cesarean delivery, and maternal death [5, 6]. Various studies have shown adverse risks in neonates, including miscarriage, congenital abnormalities, microsomia, or macrosomia with glycemic disorders, preterm birth, stillbirth, and neonatal death [7, 8]. The risk also increases in the postpartum phase with the risk of wound infection, postpartum depression, and breastfeeding rates being lower than the general population. Maternal obesity is also associated with obesity in children and adolescents [9–11].

Research on obesity in pregnancy is important to do given the high and increasing prevalence of obesity in pregnancy, as well as severe short- and long-term consequences for mothers and babies. Data obtained from research on obesity in pregnancy is expected to help determine the best strategy for preventing obesity in pregnancy and handling pregnancy in obese patients.

## 2. Materials and Methods

This research is an observational descriptive study using a cross-sectional design. The inclusion criteria are pregnant women with obesity delivered in Dr. Cipto Mangunkusumo Hospital from 2014 to 2019. The exclusion criteria for this research are incomplete medical records and having a history of previous illness before pregnancy such as hypertension and diabetes mellitus. The minimum sample size for descriptive analysis was 92 patients. Therefore, a minimum sample size of 100 subjects was determined. A total of 111 subjects were included in the study and analyzed. Based on WHO obesity divided into 3 classifications, which are obese I (30–34,9 kg/m^2^), obese II (35–39,9 kg/m^2^), and obese III/morbid obese (≥40 kg/m^2^), we analyzed the maternal and perinatal outcomes.

## 3. Results and Discussion

### 3.1. Results

In this study, there were 111 obese pregnant women who gave birth in RSCM during the study period. A total of 111 subjects were included in the study and analyzed. Analysis of the variables that are characteristic of the subject is carried out. The results of the analysis of the characteristics of the research subjects can be seen in Table 1.

Next, the characteristics of the subjects per group were compared. This comparison can be seen in Table 2.

A comparison of maternal outcomes occurred in each obese group of subjects. The maternal outcomes assessed in this study were ICU admission, gestational diabetes, preeclampsia, and premature rupture of membranes. The results of the analysis can be seen in Table 3. The preeclampsia and PROM prevalence were the highest in the obese II group.

Babies' outcomes were compared in each subject's obese group. The infant outcomes assessed in this study were gestational age, preterm birth, APGAR score of 1 minute and 5 minutes, treatment room, and birth weight. The results of the analysis can be seen in Table 4.

### 3.2. Discussion

There were 111 eligible subjects enrolled in this study. Based on the characteristics of the subjects, similar age was found between this study and similar studies, which was at an average of 31.23 ± 5.34 years. A study in Spain had similar results at an average age of 30.8 ± 6.31 years old [12]. Meanwhile, a study by Hung and Hsieh in Taipei showed that the average age of obese pregnant women was almost similar, 49.4% aged between 20 and 34 years, 41.9% over 34 years, and the remaining 0.4% was less than 20 years old [13]. In our study of maternal and perinatal outcomes in obese women, the median age was about 32 years in all subject groups, meaning the data was consistent with the characteristics shown in a similar study. This data showed that women with high-risk pregnancies become pregnant when they are overweight or obese at a rather early age in their lives.

Previously, the Institute of Medicine (IOM) lays the groundwork for the recommendation of weight gain during pregnancy. It was said that the total weight gain for overweight women was 6.8–11.3 kg, while it was 5–9.1 kg for obese women, without differentiating between obese grading whatsoever. However, the recommendation was recently argued due to the fact that the guidelines do not have any recommendation for a lower target of weight gain for women with more severe degrees of obesity. IOM also had not had any long-term study of maternal and perinatal outcomes from such recommendation [14].

As many as 86.5% of obese women in our study delivered with cesarean section and only 10.8% had undergone vaginal spontaneous labor. In RSCM Hospital from 2014 to 2019, there were 8438 births and 13,4% cases were pregnancy complicated with obesity. This is consistent with research conducted by Papachatzi studying 8,293 births, showing the risk of obese women undergoing cesarean section is significantly higher than normal BMI women [15]. After confounding factors such as diabetes and hypertension were excluded, obese subjects remained at a higher risk for cesarean delivery than control (OR 1.98, CI 1.516–2.580, *p* < 0.001). [15] In a study done by Salmon et al., a higher ratio of cesarean section was found at a higher degree of obesity (obese I 37.2%; obese II 43.4%; and obese III/morbid 52.2%) [16]. Women who experience less weight gain than those recommended by IOM have a lower risk of cesarean delivery compared to those who gain weight according to IOM (RR 0.96, CI 95% 0.94–0.97), while those with increased body weight exceeding the IOM guidelines have an overall higher risk (RR 1.11, CI 95% 1.10–1.12) [16].

A possible explanation of this finding is based on the endocrine function of adipose tissue. Adipose tissue produces hormones such as leptin and adiponectin, which play important role in metabolism, inflammatory responses, and mediating interaction between insulin and related tissues. Hormone secretion in obese women differs from women with normal BMI (adiponectin secretion is fewer in obese patients, angiotensin and TNF-*α* are higher and may cause high blood pressure and thrombosis, and also higher leptin secreted from adipose tissue may affect placental secretion). These changes would cause endothelial dysfunction responsible for worse obstetric outcomes such as preeclampsia and fetal distress, two of the most common causes of preterm labor and/or delivery through cesarean section [15].

In our study, the average parity of each group is 1 with an average pregnancy of 2 in each group. This result is similar to the obesity group data in the Laskewitz study where the mean BMI in the study group was 35.6 ± 3.0 with an average age of 28.2 ± 4.8, gestational age at delivery of 39.8 ± 0.9, and parity of 2.4 ± 1.5. Both studies also had birth weight within the normal range, albeit slightly larger than population [17].

Obesity is generally considered as a low-grade inflammatory state and associated with worse pregnancy outcomes. In a study by Laskewitz et al., parietal decidua in obese women showed significantly less M1 type macrophages (HLA-DR +, DC163−, *p* < 0.05) compared to women with normal BMI. This is likely indicating successful compensation for an increased state of inflammation in obese women. However, if this mechanism fails, pregnancy would surely be convoluted [17]. Another theory by Wilson was excess BMI during pregnancy and weight gain during pregnancy is generally believed to exacerbate the natural inflammatory condition already occurring in normal pregnancy, causing poor clinical outcomes for mother, such as increasing the risk of preeclampsia, gestational diabetes, cesarean section, and preterm labor [18]. Maternal morbidity is significantly higher in cases of obesity, but even higher when there are both gestational diabetes mellitus and obesity manifesting simultaneously. Papachatzi also said that pregnancy in obese women is at higher risk of producing poor outcomes such as gestational diabetes, preeclampsia, thromboembolism, and complications at delivery such as preterm labor, trauma, cesarean section, and pathological placental lesions [15].

In our study, 12,6% of subjects were diagnosed with gestational diabetes. There was no difference in gestational diabetes prevalence between study groups (50% in obese I, 21.4% in obese II, and 28.6% in morbidly obese, *p* > 0.05). In a study at Ontario Hospital, Canada, involving 506,483 births, the prevalence of cases of diabetes mellitus with obesity was 4.8 per 1000 births (95% CI 4.6–5.0), obesity with hypertension was 5.5 per 1000 live births (95% CI 5.3–5.7), and women with diabetes, hypertension, and obesity all together are 0.71 per 1000 births (95% CI 0.63–0.73) [21]. Similarly, according to Hung and Hsieh, overweight and obese women are more likely to have gestational diabetes mellitus (adjusted OR 2.15, 95% CI 1.80–2.56), preeclampsia (each adjusted OR 3.74, 95% CI 2.75–5.08), and dysfunctional labor (each adjusted OR 1.47, 95% CI 1.03–2.11) compared to women of normal weight [13]. In this study, the prevalence of cases of gestational diabetes with obesity was lower from a study in Canada that is 1,6 per 1000 births (14/8438); however, it differs from preeclampsia and obesity cases in our study were higher and the prevalence is 7,5 per 1000 births (64/8438).

In our study, it was found that 57.7% of subjects suffered from preeclampsia. However, there was no difference between obesity grades. Huet in his study found that there is an increased risk of preeclampsia in patients with obesity and gestational diabetes compared to patients with gestational diabetes alone. However, this risk is even greater in cases of obesity without gestational diabetes [19]. Meanwhile, Ornaghi et al. in his study found that obese women have an increased risk for the occurrence of gestational diabetes (adjusted OR, 3.18; 95% CI, 1.46–6.90), hypothyroidism (adjusted OR, 2.41; 95% CI, 1.15–5.54), and superimposed preeclampsia (adjusted OR, 2.36; 95% CI, 1.20–4.65) compared to women with normal BMI [20]. Increment in excess body weight in women with initially normal BMI is also associated with superimposed preeclampsia (adjusted OR, 3.51; 95% CI, 1.16–7.89), while an increase in excess body weight in previously obese women is associated with an increased risk of cesarean delivery (adjusted OR, 2.96; 95% CI, 1.09–5.81) [21].

In a study by Hauspurg, 7.0% of subjects had preeclampsia and 12,4% others had gestational hypertension (12.4%), much lower than the prevalence in this study. The difference in result is probably due to the sample recruited for the study. In their study, normal BMI women were also included, thus lowering the incidence of worse pregnancy outcomes. It was also found that women with postpartum hypertension tend to be older and have a BMI before pregnancy and higher postpregnancy than women with normal blood pressure [22]. In this study, there were no differences in hypertension status based on race, education, insurance status, tobacco users, or parity. Women with hypertension related to pregnancy were more likely to have hypertension at their postpartum visit compared to women whose blood pressure was normal during pregnancy (*p* < 0.001). Of all those who are overweight or obese and have preeclampsia, 80% later have hypertension (adjusted OR 2.35 (95% CI 1.63–3.41)) within one year after giving birth [23].

In our study, 18,9% of subjects suffered from PROM. Nonetheless, there was no statistical difference between groups. Maternal obesity was previously associated with an increased risk of preterm premature rupture of membranes (PPROM), after systemic inflammation. Both systemic inflation and local infection are the reasons for the increased risk of PPROM in obese women. In a previous study, it was said that they found that obese women tended to experience PPROM and labor earlier than women who were not obese, but they did not find an association between obesity and poor neonatal outcomes in this PPROM state. Gestational age at delivery was the only factor related to neonatal morbidity in their multivariate analysis [21].

The average APGAR score of infants at minute 1 in our study was 8 (0–9) and at minute 5 was 9 (0–10). In the Hung and Hsieh study, the same trend was also found with only 6 out of 267 babies (2.2%) who had an Apgar score <7 at minute 1, while at minute 5, there were no babies with an Apgar score <7 [13]. Meanwhile, in the study by Huet, 6.4% of infants of mothers with GDM without obesity had Apgar <10 at the fifth minute, 9.7% in mothers with GDM and obesity, and 10.1% in mothers with obesity alone (*p*=0.073) [19].

Babies born from subjects in this study had birth weight within the normal range, albeit slightly larger than the population. According to Hung and Hsieh, in obese mothers, babies have a tendency to be born with a large gestational period (LGA) (adjusted OR 1.86, 95% CI 1.55–2.23; and adjusted OR 2.32, 95% CI 1.67–3.20), and macrosomia (adjusted OR 1.81, 95% CI 1.24–2.64; and adjusted OR 2.51 95% CI 1.35–4.64) compared to women of normal weight [13]. Papachatzi also states that maternal obesity is associated with neonatal comorbidities such as macrosomia, low Apgar scores, and the need for care in the NICU [15]. Huet in his study found that 13.1% of infants of mothers with obesity and gestational diabetes and 13.1% of infants of obese mothers (without gestational diabetes); and 11.7% mothers with gestational diabetes only (without obese) needed ventilation [19].

Research by Bianchi et al. shows that BMI before pregnancy is associated with an increase in the birth weight of the baby, possibly because the environment is defined as metabolically rich, characterized by an increase in blood glucose and triglyceride levels in overweight and obese women. Similar to the previous explanation, high glucose levels in mothers trigger glucose transfer to the fetus which causes an increase in fetal blood glucose, increasing stimulation of insulin release, which then causes hyperinsulinemia. Furthermore, the rate of fetal glucose use will increase causing a decrease in fetal glucose, so that the gradient of transplacental glucose increases again and the rate of glucose transfer also increases. This stimulates fetal triacylglycerol formation and excessive fetal adipose tissue deposition [24].

Among 111 babies born in this study, 35,7% were treated in the NICU after delivery. A previous study had shown that neonates from obese mothers have a higher risk of being treated at the NICU. Increased morbidity (jaundice, hypoglycemia, birth defects, and congenital anomalies) in neonates from overweight or obese mothers has been reported previously which is probably the main reason that can explain this phenomenon. Neonates from obese mothers also tend to weigh more at birth. This is because obese mothers have higher glucose levels, which can penetrate the placenta and cause greater fetal growth (accompanied by hyperglycemia, hyperinsulinemia). At least 8–10% of neonates of mothers with diabetes are treated at the NICU because of hypoglycemia and perinatal distress [14]. However, higher rates of NICU patients in our study may be due to the fact that all subjects had already been in an obesity state, apart from having other complications.

In our study, 41,4% of infants were born preterm. However, there was no significant difference between study groups. Papachatzi's research shows a positive relationship between obesity and preterm labor. In that study, 15.68% of neonates from the obese mother group were born less than 37 weeks, with 5.03% born less than 34 weeks, while in control groups, only 13.4% were born less than 37 weeks. Many researchers find a positive correlation between obesity and preterm labor, while others do not support this [15].

Researchers in this study are aware of the fact that this study is significantly different than previous studies in this subject, primarily because every subject is obese, while the study groups were determined by obesity grade. This inclusion criterion alone should increase the overall complication rates in this study. Furthermore, there were 3 study groups in this study; each was according to its BMI staging. This study should present a novel finding in determining differences between BMI groups.

## 4. Conclusions

Based on this study, obese patients had a mean age of 31.23 years, mean gravida 2, parity 1, and abortion 0. Most of these patients used IUD for family planning (74.8%). There were no differences in age, parity status, and family planning methods in each group of patients with different body mass index (*p* > 0.05). Maternal characteristics are that the majority of deliveries performed cesarean delivery (86.5%), cases of gestational diabetes are more common in obese I patients (50%), preeclampsia is more prevalent in obese grade II patients (34,4%), and premature rupture of membranes (PROM) is more common in patients with obese II (52,4%). However, there was no difference in the prevalence of maternal outcomes between groups. There was a median gestational age of 37 weeks in all obesity grades, the highest percentage of preterm births owned by obese II patients (32,6%), the mean birth weight of babies tended to increase along with the weighting of the body mass index group, and NICU treatment rooms were mostly occupied from mother with obese II groups (18%). There was no difference in the first-minute and fifth-minute APGAR scores between study groups (*p* > 0.05). There were no differences in perinatal outcomes between groups.

## Figures and Tables

**Table 1:** Characteristics of subjects.

Characteristics	*N* = 111
Age (year)	31.23 ± 5.346
Body weight (kg)	85 (68–145)
Body mass index (BMI) (kg/m^2^)	36 (30–63.6)
Obese 1	39 (35.14%)
Obese 2	39 (35.14%)
Morbid obese	33 (29.72%)
Gravid	2 (1–8)
Parity	1 (0–4)
Abortion	0 (0–5)
Birth weight (gram)	2,747 ± 1,092
Gestational age (week)	37 (8–42)
Hemoglobin (g/dL)	12.3 ± 1.62
Random blood glucose (mg/dL)	105 (63–793)
APGAR score minute 1	8 (0–9)
APGAR score minute 5	9 (0–10)

*Preterm birth*	
Yes	46 (41.4%)
No	65 (58.6%)

*Diabetes mellitus*	
Yes	14 (12.6%)
No	97 (87.4%)

*Preeclampsia*	
Yes	64 (57.7%)
No	47 (42.3%)

*Premature rupture of membrane (PROM)*	
Yes	21 (18.9%)
No	90 (81.1%)

*Delivery method*	
Abortion	3 (2.7%)
Vaginal	12 (10.8%)
C-section	96 (86.5%)

*Postpartum care (mother)*	
General ward	107 (96.4%)
Intensive care unit (ICU)	4 (3.6%)

*Postnatal care (baby)*	
General ward	72 (64.8%)
NICU	39 (35,1%)

*Contraception*	
None	7 (6.3%)
IUD	83 (74.8%)
Sterilization	20 (18.0%)
Implant	1 (0.9%)

**Table 2:** Characteristics of subject groups.

Characteristics	Body mass index			
	Obese I	Obese II	Morbid obese	*p*
Age (year)	31.18 ± 5.581	31.28 ± 5.150	31.21 ± 5.453	0.930
Gravida	2 (1–8)	2 (1–5)	2 (1–7)	0.692
Parity	1 (0–4)	1 (0–4)	1 (0–3)	0.566
Abortion	0 (0–3)	0 (0–1)	0 (0–5)	0.046
Hb (g/dL)	12.1 ± 0.517	12.6 ± 0.563	12.2 ± 0.522	0.269
Random blood glucose (mg/dL)	112 (70–420)	104 (63–793)	100 (67–301)	0.188
Birth weight (1,000 gram)	2.64 ± 0.379	2.81 ± 0.314	2.80 ± 0.411	0.269
Contraception				0.542
None	2 (28.6%)	1 (14.3%)	4 (57.1%)	
IUD	30 (36.1%)	30 (36.1%)	23 (27.7%)	
Sterilization	6 (30.0%)	8 (40.0%)	6 (30.0%)	
Implant	1 (100.0%)	0 (0.0%)	0 (0.0%)	

**Table 3:** Maternal outcomes.

Characteristics	Body mass index			
	Obese I	Obese II	Morbid obese	*p*
*Delivery method*				0.422
Abortus				
Vaginal birth	1 (33.3%)	0 (0%)	2 (66.7%)	
SC	6 (50%)	3 (25%)	3 (25%)	

*Mother care*	32 (33.3%)	36 (37.5%)	28 (29.2%)	
ICU	1 (25%)	2 (50%)	1 (25%)	
General ward	38 (35.5%)	37 (34.6%)	32 (29.9%)	

*Gestational diabetes*				0.392
Yes	7 (50%)	3 (21.4%)	4 (28.6%)	
No	32 (41.4%)	36 (42.4%)	29 (16.2%)	

*Preeclampsia*				0.691
Yes	21 (32.8%)	22 (34.4%)	21 (32.8%)	
No	18 (38.3%)	17 (36.2%)	12 (25.5%)	

*PROM*				0.128
Yes	4 (19.0%)	11 (52.4%)	6 (28.6%)	
No	35 (38.9%)	28 (31.1%)	27 (30%)	

**Table 4:** Perinatal outcome.

Characteristics	Body mass index			
	Obese I	Obese II	Morbid obese	*p*
*Gestational age (week)*	37 (13–42)	37 (22–42)	37 (8–42)	0.870

*Preterm birth*				0.891
Yes	17 (37%)	15 (32.6%)	14 (30.4%)	
No	22 (33.8%)	24 (36.9%)	19 (29.2%)	

*Birth weight (gram)*	2.640 ± 379	2.810 ± 314	2.800 ± 411	0.761
APGAR score minute 1	8 (0–9)	8 (0–9)	8 (0–9)	0.785
APGAR score minute 5	9 (0–10)	9 (0–10)	9 (0–10)	0.915

*Neonatal care*				0.318
General ward	24 (21,6%)	35 (31,5%)	13(11,7%)	
NICU	15(13,5%)	20(18%)	4 (3,6%)	

## Data Availability

The data are available upon request.
